# *Siphoviridae* phage tails co-enrich with ex vivo amyloids

**DOI:** 10.64898/2026.06.17.733002

**Published:** 2026-06-18

**Authors:** Jan-Hannes Schäfer, Robert T. O’Neill, Danielle Grotjahn, Evan T. Powers, Jeffery W. Kelly, Gabriel C. Lander

**Affiliations:** a Department of Integrative Structural and Computational Biology, Scripps Research; La Jolla, CA, USA; b Department of Chemistry, Chi-Huey Wong Laboratories for Biomedical Research, Scripps Research; La Jolla, CA, USA

**Keywords:** phage, helical reconstruction, amyloidosis, cryoSPARC

## Abstract

Bacteriophages are ubiquitous in the environment and are part of the natural human microbiome. Despite their abundance, the role of the human phagome in health and disease remains poorly understood. Here, we identify phage tails in ex vivo amyloid extracts from patients with lysozyme amyloidosis (ALys) and light-chain amyloidosis (AL). Using cryo-EM analysis of the ALys dataset, automated model building, and database searches, we assigned the observed tubular assemblies to a phage tail tube protein (TTP). Although we cannot fully rule out the possibility of contamination, the presence of phage tails raises the question of whether they bind to and are co-purified with amyloid fibrils. These structures may provide further insight into the potential relationship between phage-derived assemblies and amyloid remodeling, with possible implications for future therapeutic strategies in human amyloidosis.

## Graphical Abstract

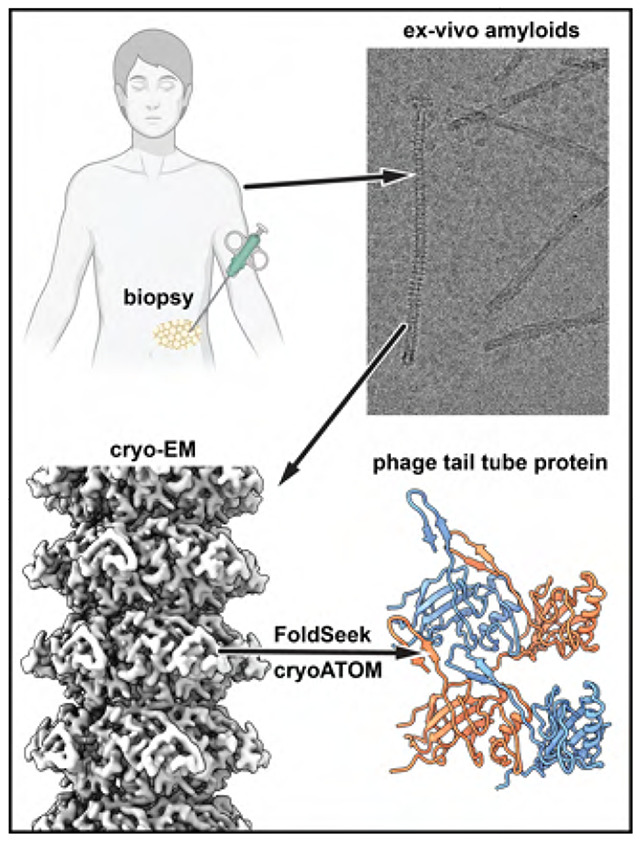

## Introduction

The human body is a large reservoir for bacterial viruses known as bacteriophages. The gastrointestinal phagome is a natural component of a healthy microbiome. Bacteriophages have been shown to translocate into the blood^18^ and tissue^10^, crossing anatomical and physiological barriers, including the gut mucosa, blood-brain, and dermal barriers^13^. Consequently, macromolecules associated with phage replication may be present throughout the human body. Among these, shed tail fibers can persist long after bacterial infection because of the high structural stability of their *β*-sheet-rich assemblies^7,12^.

Re-processing cryo-EM datasets can reveal unexpected findings, as demonstrated by the identification of vault particles in ex vivo tau samples^21^. Similarly, this approach proved informative for deposited datasets of lysozyme amyloids associated with systemic ALys amyloidosis. Here, we present the helical reconstruction of a *Siphoviridae* phage tail tube protein that were co-purified in a sample of amyloid fibrils extracted from patient tissue affected by lysozyme amyloidosis. Using the same water-based extraction approach, we also identified phage tails in cardiac amyloid extracts from a patient with light-chain amyloidosis, establishing phage tails as co-extracted assemblies in ex vivo amyloid preparations.

## Results & Discussion

### Phage tail tube protein co-enriches with ex-vivo ALys amyloids

**Figure 1.**
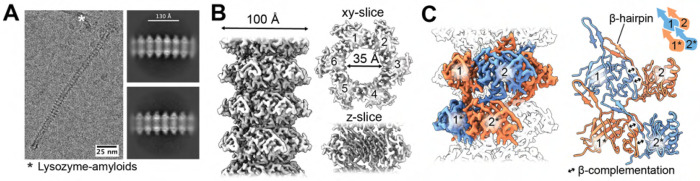
Reconstruction of a phage tail-tube protein from ex vivo lysozyme amyloids. (**A**) Micrograph from EMPIAR-11785 showing 2D class averages of a non-amyloid tubular fibril; scale bar: 25 nm. (**B**) 3.5 Å helical reconstruction of 10 nm C6-symmetric tubes with a 3.5 nm inner diameter, displayed along the helical axis and as xy- and z-slices. Numerals indicate individual protofilaments. (**C**) Cryo-EM reconstruction color-coded by subunit packing. Inter-protofilament contacts through *β*-complementation are indicated by black double arrows.

During re-processing of an ex vivo lysozyme variant D87G^16^ dataset (EMPIAR-11785), associated with ALys, we identified non-amyloid fibrillar assemblies by 2D classification (Fig. 1A). Approximately one-third of micrographs contained these distinct fibrillar structures. Helical refinement using a rise of 37.23 Å and twist of 14.17° yielded a 3.5 Å resolution reconstruction of 10 nm diameter tubes with a C6-symmetric protofilament arrangement and an inner diameter of 3.5 nm (Fig. 1B, S1).

**Figure 2.**
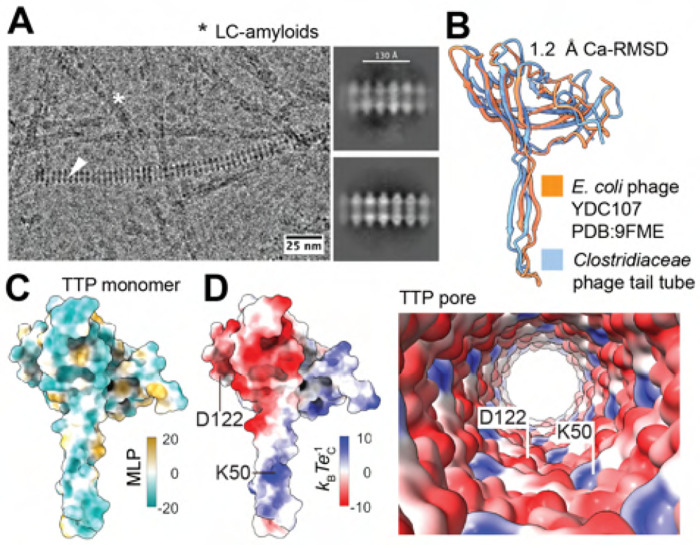
Phage tails are structurally conserved and present in ex vivo amyloid extracts. (**A**) Micrograph of light-chain amyloids (asterisk) and phage tails (arrow). Scale bar 25 nm. 2D class averages of phage tail fibrils, scale bar 130 Å. (**B**) Structural comparison of the *E. coli* phage YDC107 tail tube protein (PDB: 9FME, orange) and the presented *Clostridiaceae* phage tail tube protein described here (blue). Backbone root mean square deviation (RMSD), 1.2 Å. (**C**) Surface property analysis of the *Clostridiaceae* phage tail protomer, showing molecular lipophilicity potential (MLP) and (**D**) electrostatics of the TTP monomer and the assembled filament, viewing through the pore. Solvent accessible residues D122 and K50 are annotated.

To identify the assembly, we used automated model building with CryoAtom^26^, using the human proteome (UP000005640) as a reference database. No convincing matches were identified, arguing against our initial interpretation that the fibrils represented stacks of common amyloid-associated proteins, such as serum amyloid P component (SAP), or intermediates of lysozyme amyloid formation. We then used the fitted poly-alanine backbone trace from automated model building as input for Foldseek^29^ to identify proteins with similar folds, which indicated the presence of phage tail tube proteins (TTPs) from the *Caudovirales* order, consistent with the *Siphoviridae* morphology of tailed phages. Because the global resolution was limited to approximately 3.5 Å, the structure-to-sequence assignment remains probabilistic and relies in part on alignment of aromatic residues. Although many common phage proteomes are known, we cannot exclude the possibility that the observed phage tail originates from an unclassified species. Manual inspection of the 10 Foldseek targets with the highest confidence scores against the AlphaFold database (AFDB50, Fig. S3) supported assignment of the structure to a *Clostridiaceae* phage tail tube protein (D9SPF2, Fig. 1C; Fig. S3). Notably, the 2D class averages and reconstruction lacked decorating domains such as Ig-like domains, which may reflect proteolytic degradation or point to TTPs without Ig-like domains. Each protomer within the hexameric TTP stacks contains a conserved *β*-sandwich domain, that lines the central genome delivery channel and an extended *β*-hairpin, involved in inter-stack interactions (Fig. 1C, 2C). Surface electrostatics and hydrophobicity analyses are consistent with nucleotide transit through the polar tail tube channel (Fig. 2C–D). Comparison of the assigned *Clostridiaceae* phage TTP with the *E. coli* phage YDC107 TTP, which was reconstructed from bacterially contaminated buffers, revealed strong structural conservation, with a backbone RMSD of 1.2 Å (Fig. 2B).

### Are shed phage tails frequently co-enriched with amyloids?

We next asked whether phage tails are frequently co-enriched with ex vivo amyloids. To address this, we analyzed cardiac light-chain amyloids (AL), which cause systemic amyloidosis and arise from the overproduction of immunoglobulin light-chain fragments. Clinically, AL amyloidosis can affect the heart, digestive tract, kidneys, nervous system, and soft tissue^1^. Following an established water-based extraction protocol, we enriched light-chain amyloids from the apex of a heart explanted from an AL amyloidosis patient heart for cryo-EM. Using reference-based particle picking, we also identified phage tails in the AL dataset as well (Fig. 2A). Whereas nearly one-third of ALys micrographs contained observable phage tails, only approximately 5% of our AL micrographs contained phage tails. These percentages were estimated by linking particles assigned to phage-tail 2D class averages back to their original micrographs. Because of the limited number of particles in the AL dataset, we were unable to reconstruct the AL-associated phage tails. Using the 2D class averages of the proposed phage tails to generate an average power spectrum in Fourier space, we estimated a helical rise of approximately 37 Å, closely matching the ALys phage tail tubes with a rise of 35 Å (Tab. S1, Fig. S4B), further supporting our assessment of co-enriched phage tails in ex vivo amyloid extracts.

### Do phages naturally bind to amyloids?

One possible explanation for the co-enrichment of phage tails with amyloids is external contamination during sample preparation, but in our laboratory, phages have only been observed in the AL patient sample. Alternatively, phage tails may be naturally present in the extraction source and enriched through affinity- or avidity-based interactions with amyloid fibrils. Support for amyloid-phage interaction centers on the putative coevolution of bacterial amyloids and phages. In this evolutionary “arms race,” bacteria and phages have developed mechanisms for both defense and counter-defense, including interactions with amyloid structures. Functional amyloids are widely found in bacterial biofilms, where they evolved to serve roles as virulence factors, as defense mechanisms, and for structural support^4^. These amyloid functions also include the sequestration of bacteriophages^27^. For example, the most widely studied functional amyloid is CsgA and it is a major component of bacterial biofilms. Curli fibers (amyloid fibers composed of CsgA) help bacteria adhere to surfaces, act as structural supports, and act as a ‘sticky net’ to sequester phages^14,19^. In addition, functional and pathogenic amyloids interact with many of the same macromolecules of the extracellular matrix^24^, supporting the possibility that functional amyloids and the systemic lysozyme and light-chain fibrils studied here share related physical properties and binding partners. In this context, a general amyloid interaction motif (GAIM) has been identified in the M13 phage capsid protein g3p (N1-N2) (amino-terminal domains), which recognizes amyloid folds^17^. GAIM-Fc complexes can induce amyloid conversion into amorphous aggregates, including the presented light-chain amyloids^3^, although clinical exploration of GAIM-Fc has not progressed beyond Phase 1b clinical trials (NCT03879278, NCT03610035). Although g3p and TTPs share structural features (Fig. S4A), whether the resolved TTP contains a GAIM-like determinant that mediates affinity-driven interactions with ex vivo amyloids remain to be tested. Although our data are consistent with affinity-based co-purification of amyloids and TTPs, we cannot exclude enrichment driven by shared physical properties during the sedimentation-based extraction protocol. Further characterization will be needed to distinguish between these possibilities, but such experiments are beyond the scope of this study.

Overall, re-processing heterogeneous cryo-EM datasets from public repositories such as EMPIAR revealed a phage tail tube protein in ex vivo amyloid extracts. These findings highlight the value of re-examining complex biological specimens and raise questions about the sparsely explored human phagome and its potential interactions with the human proteome.

## Material & Methods

### Amyloid light-chain extraction

This study, conducted with anonymized ex vivo human cardiac tissue samples obtained by a generous gift from Protego Bio from a donor who provided informed consent, was approved by the Institutional Review Board of Scripps Research (protocol No.: IRB-24-8382, continuing IRB-16-6864 and IRB-12-5994). Light-chain amyloids were extracted from human heart tissue using a water-based protocol^2^. Briefly, 125-250 mg of cardiac tissue from the apex was cut, washed three times with 0.5 mL Tris-Calcium buffer, and incubated overnight with *C. histolyticum* collagenase (0.5 mg/mL) at 37 °C. The sample was pelleted at 3100 x g for 30 min at 4 °C, and the supernatant was discarded. The pellet was resuspended in 0.5 mL Tris-EDTA, centrifuged, and the supernatant was discarded. After 10 total extraction cycles, the pellet was resuspended in 0.5 mL ice-cold water and stored at 4 °C.

### Cryo-electron microscopy of cardiac light-chain amyloids

UltraAuFoil 200 mesh R 2/2 grids were glow-discharged under vacuum for 30 s at 15 mA in a Pelco 311 easiGlow 91000 glow discharge cleaning system (Ted Pella). 3.5 μl sample was applied to the front of the grid and 0.5 μl to the back, incubated for 1 min, and backblotted with Whatman 1 filter paper for 4-6 s after the liquid spot on the filter paper stopped spreading. Grids were manually plunge-frozen in a 4 °C cold room at >95% humidity. AutoGrids were assembled and immediately screened.

Cryo-EM data were collected on a Talos Arctica TEM (Thermo Fisher) operating at 200 keV. Movies were recorded using a Falcon 4i direct electron detector (Thermo Fisher) at a nominal magnification of 150,000, corresponding to a pixel size of 0.94 Å . Movies were saved in the electron-event representation (EER) format and recorded with a total electron exposure of 50 e^−^ per Å^2^. All datasets were collected automatically using EPU (v.3.9, Thermo Fisher) with a defocus range of −0.8 to −2.0 μm. EPU’s fast exposure navigation was used to collect data with an 8 μm image shift. All datasets were processed using cryoSPARC (v.4.6).

For preprocessing, movies were dose-fractionated into 40 frames and motion corrected using patch-based motion correction, followed by patch-based CTF estimation in cryoSPARC Live. Micrographs with CTF fits worse than 6 Å and astigmatism greater than 600 Å were discarded. The remaining accepted micrographs were used for further processing. Perdataset micrograph statistics are compiled in Table S1.

### Processing of tubular filaments in EMPIAR-11785

From the 2,013 processed micrographs in EMPIAR-11785, approximately 30% contained non-amyloid segments and were selected for further processing. To increase the number of particles, filaments were traced using a diameter range of 40-80 Å without templates and with 0.5 x Gaussian blur. Segments were extracted in a 208-pixel box, Fourier-cropped to 100 pixels, and subjected to two rounds of 2D classification with an initial class uncertainty factor of 4 and hard classification during the final iteration. A total of 30,000 selected segments were re-extracted with recentering in a 256-pixel box without binning. Helical refinement was performed without symmetry using a cylindrical model and a 60° tilt search-range. The resulting reconstruction was used as input for real-space helical indexing in HI3D^5^ with default parameters, suggesting a twist of 13.74° and a rise of 37.93 Å. These values were used in helix refine with non-uniform refinement (NUR) enabled. To test for higher-order symmetry, Phenix’s symmetry search tool *phenix.map_symmetry*^20^ was used and identified the highest correlation with C6 point-group symmetry. After another round of 2D classification, 21,000 segments were used in helical refinement with C6 symmetry, a rise of 38 Å and a twist of 13.7°. Helical indexing with the new C6 point group and higher-resolution map suggested a rise of 37.19 Å and twist of 14.21°. The final helical refinement with a 20° tilt search-range resulted in a 3.5 Å reconstruction (FSC 0.143). The map was sharpened automatically within cryoSPARC using a B-factor of −56 Å^2^. The full processing workflow and validation are shown in Fig. S1, Fig. S2 and Table S1.

### Model Building and Refinement

Map resolvability was improved through postprocessing in EMready2^8^ and used for automated model building in CryoAtom^26^. The initial backbone trace was used to mask individual fibril subunits and submitted to Foldseek^29^. The best 20 hits from the AFDB50 were utilized as a database for sequence-based model building in CryoAtom. After three iterations, the top 10 hits were manually inspected in Coot^11^ for map-model fits of aromatic side chains using AlphaFold^15^ models. Further validation was performed using the Q-score^23^ for residue-wise quality estimation. The best model was further refined using ISOLDE^9^ and Phenix^20^. Results were visualized using ChimeraX^22^.

### Comparative power spectrum analysis

2D class averages of ALys and Light-chain phage fibrils from cryoSPARC were opened with the *relion_display* function in RELION-5^6^ and converted to their power spectrum. The first layer line distance was manually measured from the equator and converted to the helical rise value according to established protocols^25^.

## Supplementary Material

1

## Data Availability

All re-processed datasets from the Electron Microscopy Public Image Archive (EMPIAR) were deposited in the Electron Microscopy Data Bank (EMDB) and Protein Databank (PDB). The datasets from Table S1 are available using the following EMDB accession numbers with their respective EMPIAR-ID: Phage-tail protein (PDB-11OK, EMD-72138, EMPIAR-11785).
