# Supplementary material for: Siphoviridae phage tails co-enrich with ex vivo amyloids
Source: bioRxiv. 2026 Jun 18:2026.06.17.733002. Preprint. [Version 1] doi: 10.64898/2026.06.17.733002 (PMC13308092; doi:10.64898/2026.06.17.733002)
Supplement: 1 [file NIHPP2026.06.17.733002v1-supplement-1.pdf]

bioRxiv preprint doi: https://doi.org/10.64898/2026.06.17.733002; this version posted June 18, 2026. The copyright holder for this preprint (which was not certified by peer review) is the author/funder, who has granted bioRxiv a license to display the preprint in perpetuity. It is made available under a CC-BY 4.0 International license.

Supporting Information

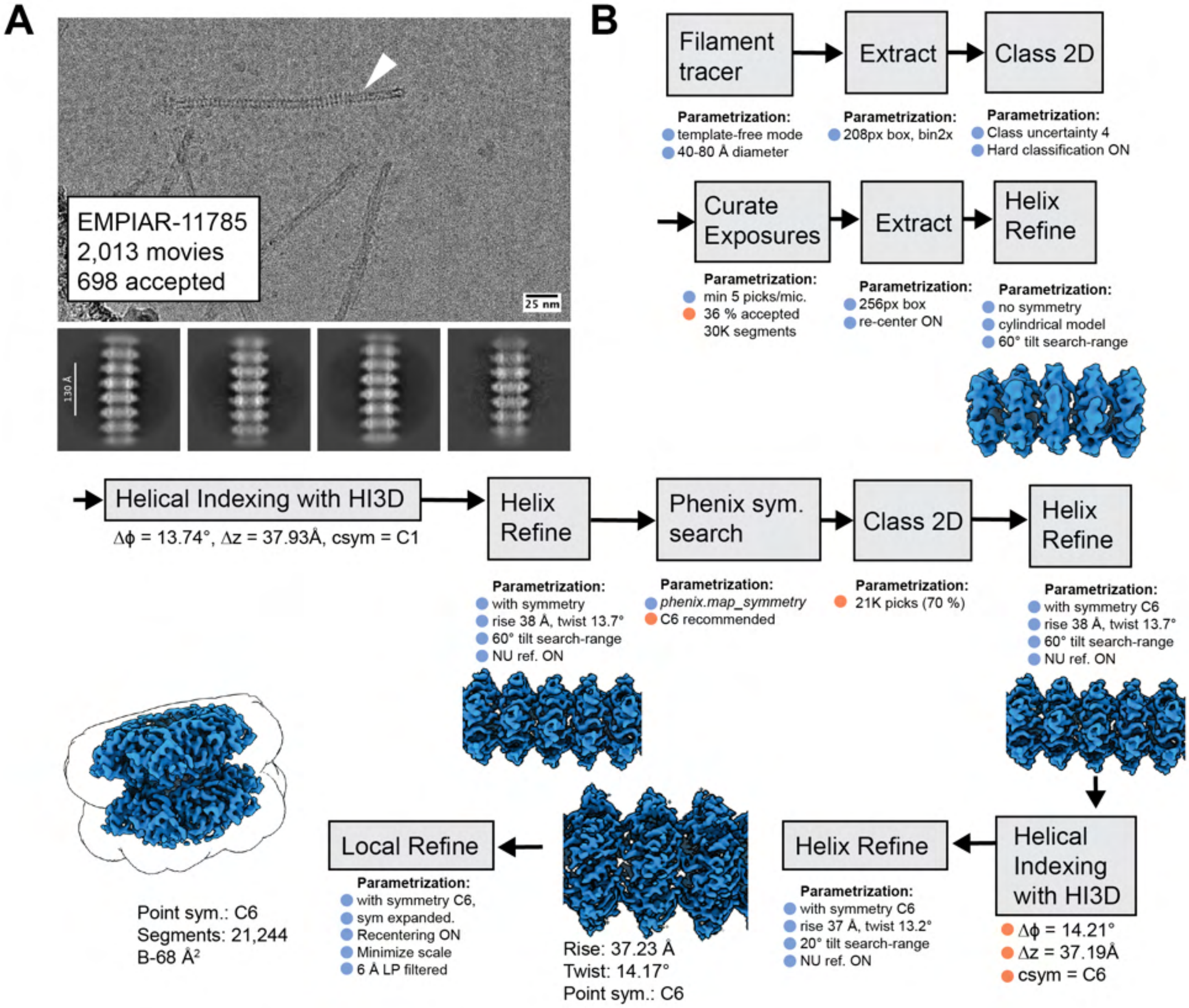

Figure S1. Reconstruction of a *Siphoviridae* phage tails. (**A**) Micrograph and 2D class averages from EMPIAR-11785 positive for the phage tails (white arrow). 25 nm scale bar on micrograph and 130 Å scale bar on 2D class averages. (**B**) Processing workflow for helical reconstruction.

bioRxiv preprint doi: https://doi.org/10.64898/2026.06.17.733002; this version posted June 18, 2026. The copyright holder for this preprint (which was not certified by peer review) is the author/funder, who has granted bioRxiv a license to display the preprint in perpetuity. It is made available under a CC-BY 4.0 International license.

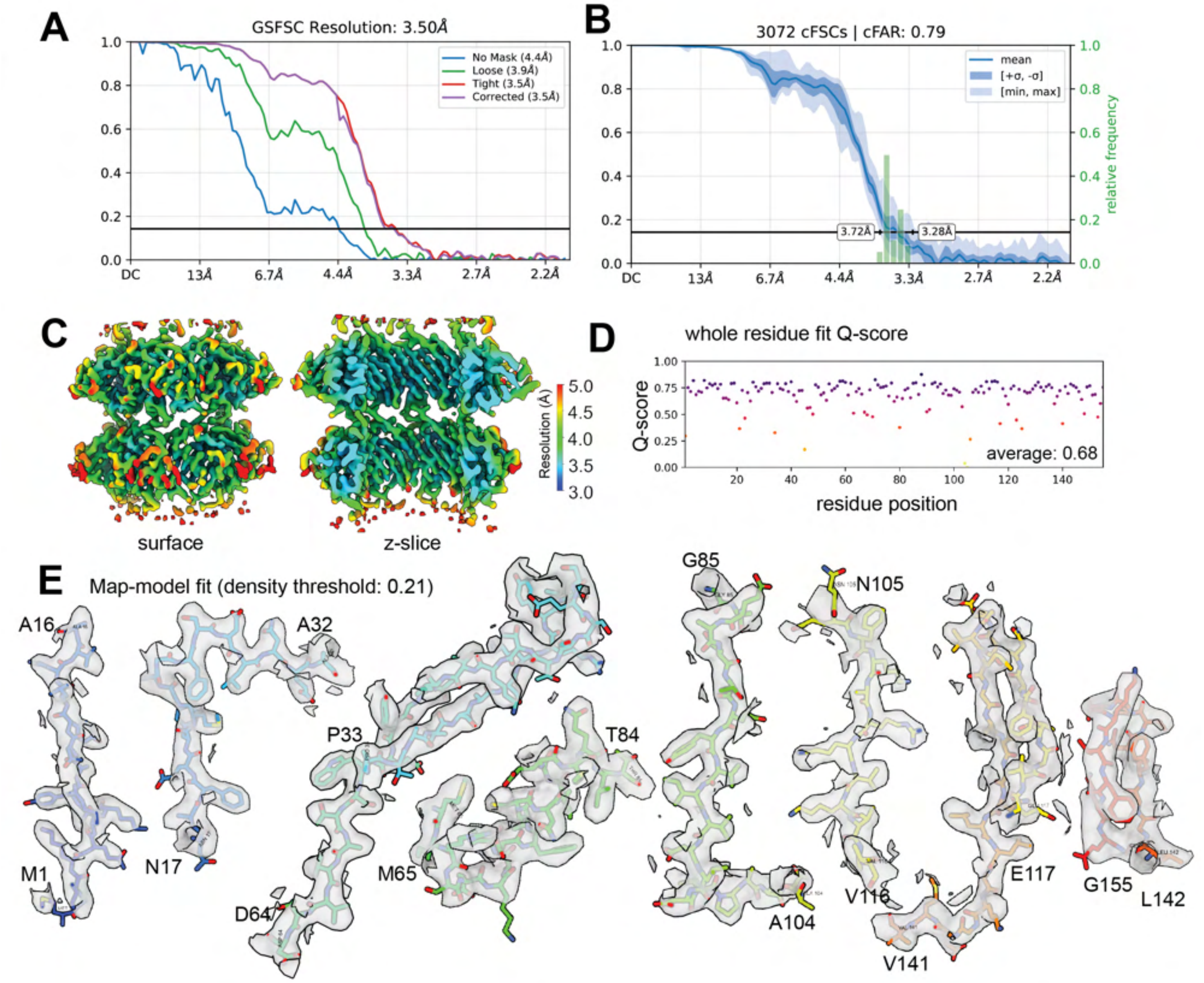

Figure S2. Validation of a *Siphoviridae* phage tail reconstruction. (**A**) Gold-Standard FSC resolution at threshold 0.143. (**B**) Orientation diagnostics, including Conical FSCs (cFSC) and Conical FSC area ratio plots (cFAR). cFAR ¡ 0.5 indicates anisotropic reconstruction. (**C**) Local resolution estimates with surface- and z-slice representation. (**D**) Atom resolvability analysis using the whole residue Q-score plot and a global average of 0.68. (**E**) Map-model fit of the entire monomer, density threshold at 0.21.

bioRxiv preprint doi: https://doi.org/10.64898/2026.06.17.733002; this version posted June 18, 2026. The copyright holder for this preprint (which was not certified by peer review) is the author/funder, who has granted bioRxiv a license to display the preprint in perpetuity. It is made available under a CC-BY 4.0 International license.

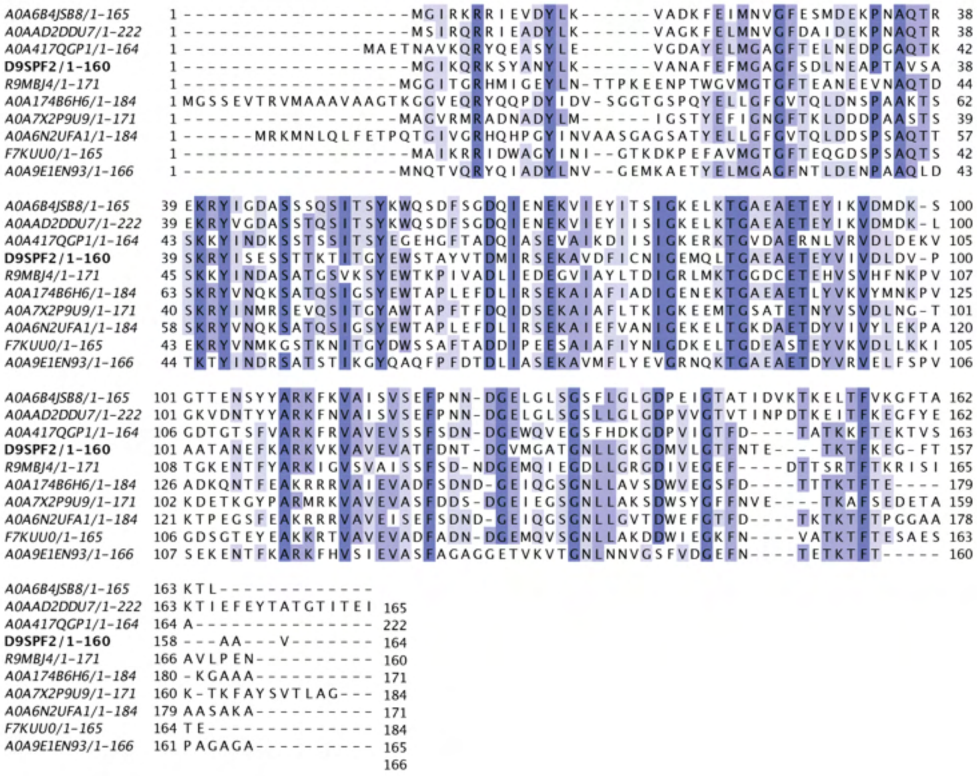

Figure S3. Multiple-sequence alignment. Final 10 sequence hits from FoldSeek[29] using the AFDB50 database. Sequences are color-coded by conservation (sequence identity) and aligned using Clustal-Omega within Jalview[28]. The best map-to model fit is highlighted in bold (Uniprot D9SF2, *Clostridiaceae* phage tail protein).

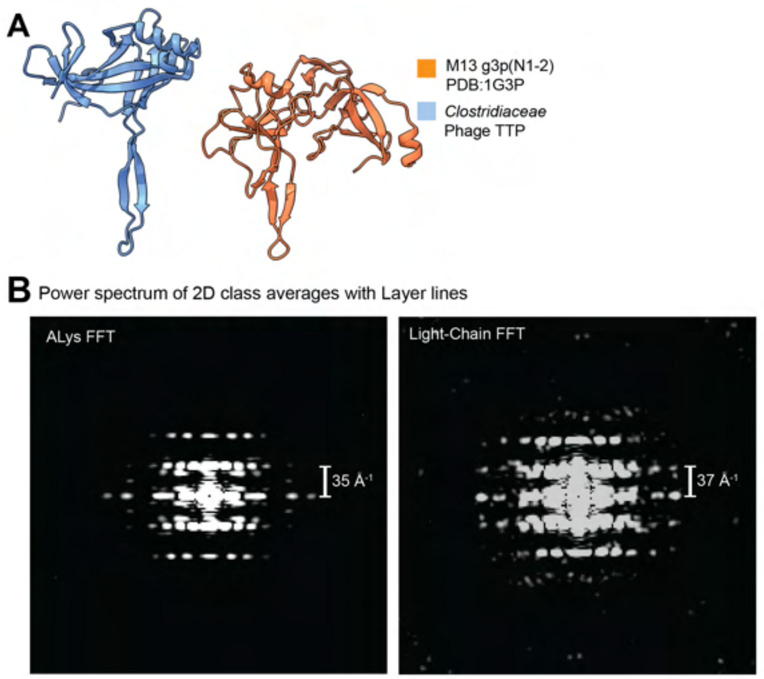

Figure S4. (**A**) Comparison of the resolved Clostridiaceae phage TTR (blue) and M13 Phage N-terminal domain of minor coat protein g3p (orange, PDB: 1G3P). (**B**) Comparison of power spectra of the ALys (left) and Light-chain (right) 2D class averages using the *relion_display* function in RELION-5[6]. The annotated layer-line approximates the helical rise of the helical assembly.

bioRxiv preprint doi: https://doi.org/10.64898/2026.06.17.733002; this version posted June 18, 2026. The copyright holder for this preprint (which was not certified by peer review) is the author/funder, who has granted bioRxiv a license to display the preprint in perpetuity. It is made available under a CC-BY 4.0 International license.

Table S1. Cryo-EM data collection and image processing of phage tails.

	**Phage Tail**
EMDB ID	72138
EMPIAR ID	11785
PDB ID	11OK
Microscope	Krios
Camera	K2 Quantum
Magnification (nom.)	130,000
Voltage (keV)	300
Total dose (e⁻/Å^2)	49
Exposure rate (e⁻/px/s)	5.9
Frames per movie	40
EER Fractions	NA
Pixel size (Å/px)	1.04
Defocus range (µm)	−1.0 to −2.0
Recorded movies	2,013
Processing software	CS (v.4.7)
Final particle images	21,244
Symmetry imposed	C_6
Helical twist (°)	+14.17
Helical rise (Å)	37.23
Res. (FSC 0.143, Å)	3.5
Local res. range (Å)	3.3–3.7
cFAR	0.79
Sharpening B-factor (Å^2)	−68
Initial model	AlphaFold (D9SPF2)
Refinement	Coot/ISOLDE/Phenix
FSCmap-to-model(0.5) (Å)	3.8
MolProbity score	1.70
Clash Score	7.89
Composition	
Chains	12
Atoms	27,756
Protein residues 1,848	
Bonds (R.M.S.D.)	
Length (Å)	0.003
Angles (°)	0.771
B-factors (min/max/mean)	
Protein residues	64.48/87.08/73.68
Ramachandran plot (%)	
Favored	96.05
Allowed	3.95
Outliers	0.00
Rotamer outliers (%)	0.00
Q-score	0.68
EMRinger score	3.20
